# OA14.03. Effects of yoga on sleep, mood, and related outcomes in older women with Restless Legs Syndrome: a nested randomized controlled trial (RCT)

**DOI:** 10.1186/1472-6882-12-S1-O55

**Published:** 2012-06-12

**Authors:** K Innes, T Selfe, G Alexander, C Bourguignon, A Taylor, I Hinton

**Affiliations:** 1 West Virginia University, Morgantown, USA; 2 Texas Christian University, Dallas, USA; 3 University of Virginia, Charlottesville, USA

## Purpose

Restless Leg Syndrome (RLS), a common sleep disorder, has been linked to increased cardiovascular disease (CVD) risk, key components of the metabolic syndrome, and is associated with significant societal and economic burden. Current treatments are often ineffective and can have serious side effects, highlighting the need to investigate promising new non-pharmaceutical approaches. In this nested RCT, we examined the effects of a gentle yoga program vs. an educational film program on sleep, mood, perceived stress, blood pressure, and heart rate in women at risk for CVD.

## Methods

Participants were drawn from a larger trial regarding the effects of yoga on CVD risk profiles. Seventy-five overweight, sedentary postmenopausal women aged 45-79 years were randomized to receive either an 8-week yoga (n=38) or educational film (n=37) program. Main outcomes assessed pre- and post-treatment included measures of sleep (Pittsburgh Sleep Quality Index), stress (Perceived Stress Scale), mood (Profile of Mood States, State-Trait Anxiety Scale), blood pressure, and heart rate. Participants completed an RLS screening questionnaire at baseline. Twenty (27%) of the 75 women met standardized diagnostic criteria for RLS (n=10 yoga, 10 film group).

## Results

At baseline, women with RLS demonstrated significantly poorer sleep quality, higher blood pressure, and greater likelihood of having a history of depression than those without RLS. Among participants with RLS, those assigned to the yoga group demonstrated significantly greater improvement than did controls in sleep quality, perceived stress, mood, anxiety, and blood pressure (p≤0.05). Among participants with RLS and insomnia (PSQI>5) at baseline, 86% of the yoga group (6/7) vs. 30% of controls (3/10) had scores in the normal range post-intervention (p=0.03). Adjustment for treatment expectancy did not materially alter these findings.

## Conclusion

These preliminary findings suggest that yoga may offer a safe, effective intervention for reducing sleep and mood disturbance, perceived stress, anxiety, and blood pressure in women with RLS.

